# Zero-Order Kinetics Release of Lamivudine from Layer-by-Layer Coated Macromolecular Prodrug Particles

**DOI:** 10.3390/ijms252312921

**Published:** 2024-12-01

**Authors:** Tomasz Urbaniak, Yauheni Milasheuski, Witold Musiał

**Affiliations:** Department of Physical Chemistry and Biophysics, Pharmaceutical Faculty, Wroclaw Medical University, Borowska 211, 50-556 Wrocław, Poland; tomasz.urbaniak@umw.edu.pl (T.U.); eugeniusz.miloszewski@gmail.com (Y.M.)

**Keywords:** core-shell microparticles, zero-order release kinetics, layer-by-layer coating, polyelectrolyte shells, macromolecular prodrugs, ring-opening polymerization, prolonged lamivudine release

## Abstract

To reduce the risk of side effects and enhance therapeutic efficiency, drug delivery systems that offer precise control over active ingredient release while minimizing burst effects are considered advantageous. In this study, a novel approach for the controlled release of lamivudine (LV) was explored through the fabrication of polyelectrolyte-coated microparticles. LV was covalently attached to poly(ε-caprolactone) via ring-opening polymerization, resulting in a macromolecular prodrug (LV-PCL) with a hydrolytic release mechanism. The LV-PCL particles were subsequently coated using the layer-by-layer (LbL) technique, with polyelectrolyte multilayers assembled to potentially modify the carrier’s properties. The LbL assembly process was comprehensively analyzed, including assessments of shell thickness, changes in ζ-potential, and thermodynamic properties, to provide insights into the multilayer structure and interactions. The sustained LV release over 7 weeks was observed, following zero-order kinetics (R^2^ > 0.99), indicating a controlled and predictable release mechanism. Carriers coated with polyethylene imine/heparin and chitosan/heparin tetralayers exhibited a distinct increase in the release rate after 6 weeks and 10 weeks, respectively, suggesting that this coating can facilitate the autocatalytic degradation of the polyester microparticles. These findings indicate the potential of this system for long-term, localized drug delivery applications, requiring sustained release with minimal burst effects.

## 1. Introduction

Control over the drug release mode and rate is one of the most critical aspects considered during the design of drug delivery systems (DDS). Micro- and nanodrug carriers, due to their large surface area, often exhibit burst release—a phenomenon frequently observed in drug release studies—which can negatively impact the system’s therapeutic performance [1]. In the last few decades, targeted drug delivery via colloidal carriers that accumulate in the tissue of interest has been a widely researched area in pharmaceutical sciences. Numerous approaches have been proposed to selectively deliver incorporated drugs, with the most frequently employed methods involving delivery through dedicated ligands and stimuli-responsive release. Passive targeting strategies, such as cytostatic drug accumulation through the enhanced permeability and retention effect in tumor-targeted delivery, are also proposed as promising strategies to reduce unwanted side effects [2]. After systemic administration, most carriers will circulate for some time until passive or active targeting mechanisms lead to their accumulation in the tissue of interest. Therefore, a burst release observed in the initial phases of carrier exposure to aqueous media is particularly unfavorable, as it may significantly decrease the amount of drug delivered selectively. From a pharmacokinetic perspective, the burst release effect observed in both traditional drug forms and novel colloidal carriers is highly undesirable for maintaining safe and therapeutic serum drug levels [3]. Precise control over the drug release profile is one of the most crucial requirements for drug carriers, to ensure the safety and efficiency of the therapeutic process.

The choice of carrier material that exhibits a high affinity to the incorporated drug is the most basic strategy to slow down the initial release from particulate DDS. Nevertheless, in virtually all systems where the release is diffusion-driven, the profiles will follow non-zero-order kinetics, well-described by Higuchi and Korsmeyer-Peppas’ equations [4]. Only in cases where highly hydrophobic drugs are incorporated into hydrophobic matrices, such as estradiol in PLGA microparticles, can release profiles that are close to linear be achieved [5]. For hydrophilic drugs, controlling the release is a more challenging task. In traditional dosage forms, the approach based on osmotic pumps is the most widely employed strategy to provide zero-order kinetics. However, for colloidal carriers and drug-releasing polymeric implantable devices, achieving zero-order kinetics is considered non-feasible [6].

One of the strategies employed to modify the pharmacokinetic fate of an administered drug is the use of prodrugs. Prodrugs are dormant complex compounds that undergo metabolic alterations, leading to the formation of active drug forms. There are multiple examples of marketed prodrugs that exhibit beneficial features compared to the administration of the corresponding active substance, such as improved bioavailability (e.g., oseltamivir), reduced systemic side effects (e.g., capecitabine), and reduced risk of drug abuse (e.g., lisdexamfetamine). These favorable properties can be achieved through various chemical modifications, including esterification, amidation, redox reactions, or the formation of carbamates or carbonates [7]. The introduction of larger, macromolecular components into the structure of a bioactive molecule leads to the formation of so-called drug–polymer conjugates [8]. These conjugates can essentially serve as prodrugs, assuming that at some point after administration, the free drug molecule is detached from the macromolecular construct. Depending on the properties of the macromolecular component, such materials can be formed into various DDSs, including micro- and nanoparticles, allowing desired release profiles to be achieved. In traditional oral drug formulations, coatings and semipermeable membranes are commonly used to modify drug release profiles. However, applying this approach to micro- and nanoscale drug delivery systems (DDS) is often challenging. Instead, alternative methods, such as layer-by-layer (LbL) coating, can be employed to encapsulate micro-objects with polymer shells, thereby potentially altering the drug release profile from the particle core. Due to the mild coating conditions and the wide variety of available polyelectrolytes, this versatile method is extensively used in the biomedical field [9]. Over the past three decades, significant advancements have been made in understanding the molecular-scale phenomena involved in the assembly of LbL systems, enabling the correlation of these processes with unique film properties that hold considerable practical value [10,11,12].

**Figure 1:**
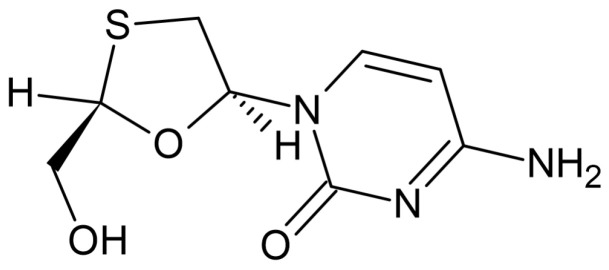
Chemical structure of lamivudine employed as ring-opening polymerization initiator.

In this work, we investigated the release profile of lamivudine (LV) from microparticles formulated from a macromolecular prodrug obtained through ring-opening polymerization. LV is a nucleoside reverse transcriptase inhibitor used primarily in the treatment of HIV and hepatitis B infections (Figure 1).

LV acts by incorporating into the viral DNA during replication, leading to premature chain termination and inhibition of viral reverse transcriptase. This mechanism effectively reduces viral replication, helping to control infection and limit disease progression. For HIV, lifelong therapy is required, and prolonged, controlled delivery of antiviral drugs offers significant benefits in improving both the safety and efficacy of treatment [13]. The feasibility of using nucleoside analogues in ring-opening polymerization has been previously demonstrated, establishing LV as a promising candidate for a prodrug component [14]. Other examples of successful drug conjugation during ring-opening polymerization include paclitaxel-, doxorubicin-, and docetaxel-based polyester macromolecular prodrugs [15,16]. The fabricated carriers, derived from the synthesized prodrug, were coated with two variants of layer-by-layer (LbL) shells composed of chitosan (CHIT), polyethyleneimine (PEI), and heparin (HEP). The potential role of these coatings as diffusion barriers influencing LV release profiles was investigated.

## 2. Results and Discussion

### 2.1. Macromolecular Prodrug Synthesis

**Figure 2:**
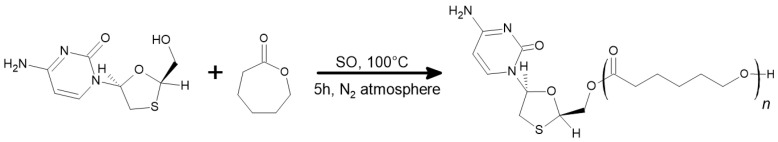
Scheme of ring-opening polymerization reaction initiated by lamivudine.

According to the proposed mechanism of ε-caprolactone (ε-CL) ring-opening polymerization catalyzed by stannous octoate (SO), the catalyst acts as an oxygen-coordinating Lewis acid, which facilitates a nucleophilic attack of alcohols on the carbonyl carbon of the monomer. This leads to ring-opening, and the further propagation of polymer through a repeated attack of the growing chain-end on another one of the monomer molecules. As a result, alcohol serving as a reaction initiator is covalently bound to the polymer chain via an ester bond (Figure 2) [17].

**Figure 3:**
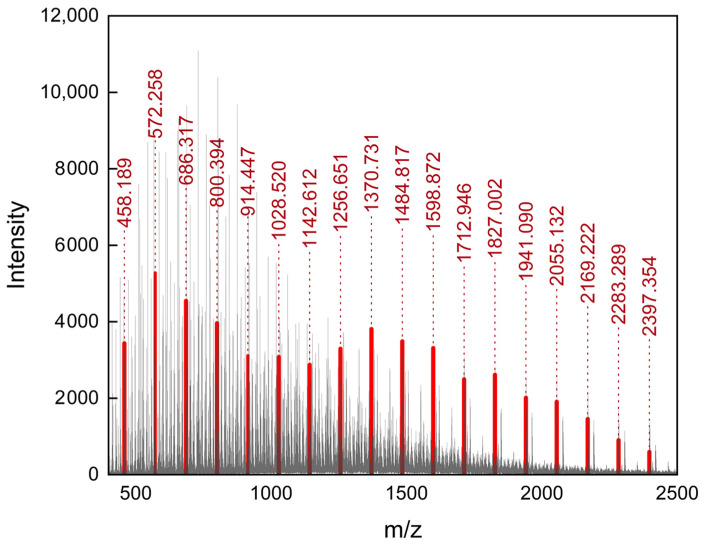
Mass spectra of LV-initiated ring-opening polymerization product with ionic distributions subset attributed to LV-PCL macromolecular prodrug molecules (marked in red).

LV is one of the active pharmaceutical ingredients which, due to the presence of a hydroxyl group, may be applied as a reaction initiator [18]. The prodrug obtained as a result of the reaction is a mixture of differently sized macromolecules, which are reflected in the complexity of the mass spectra obtained with ESI-ToF MS (Figure 3).

**Figure 4:**
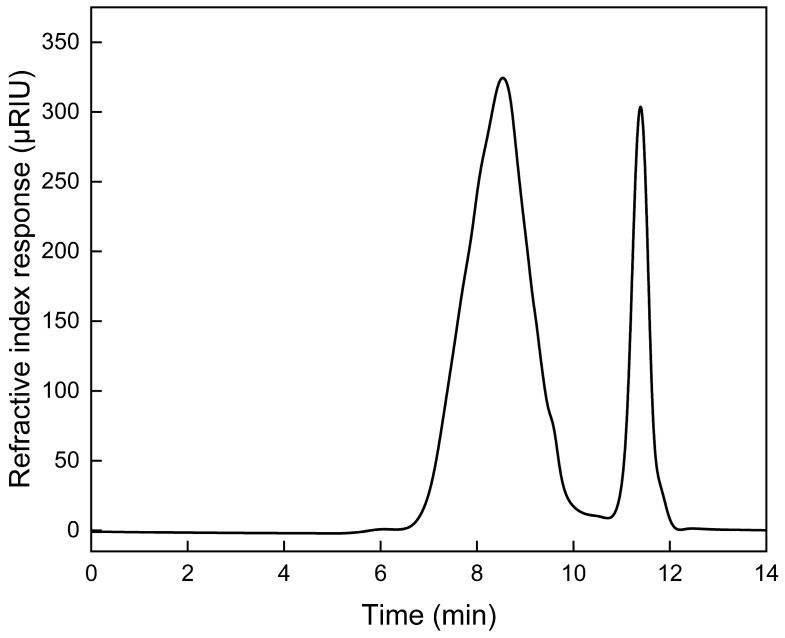
GPC chromatogram of LV-initiated ring-opening polymerization product.

In the ESI-ToF MS method, the ionization of analyzed molecules is achieved through rapid evaporation of the fine droplet mist of the polymer solution. Some of the mixture constituents, mainly those of heavier chains, may experience ion suppression due to poor solubility, which restricts their transfer from the droplet to gas phase [19]. Additionally, the high molecular weight of the analyzed macromolecules may result in the formation of multiply charged ions, significantly increasing the number of isotopic distributions present in the spectra [20]. Those factors limit the amount of possible information which can be derived from the obtained data. In the presented spectra, the analysis of isotopic distributions revealed the presence of an ionic distributions set, which can be described by the general formula of C_(8+(n×6)_H_(11+(n×10)_O_(3+(n×2))_N_3_S_1_ (Figure 3, marked in red). This formula represents the LV molecule coupled with n PCL monomer units, where n ranges from 2 to 19, as depicted in Figure 2, as the product of the reaction. The average matching error was 0.0039 ± 0.0016 Da. The high intensity of this isotopic distribution set indicates that LV-PCL was the main component of ions obtained through the ESI of the polymerization product solution. A more comprehensive spectral analysis of an analogous prodrug obtained under different reaction conditions was described in a previous study by our group [21]. The bimodal intensity distribution of these isotopic patterns in the mass spectra suggests the presence of two main, overlapping chain subpopulations of conjugates. This observation did not correspond to the GPC chromatogram of LV-PCL, where a symmetrical peak, derived from the polymer, was observed at an elution time of 8.5 min (Figure 4).

The numbers comprising the average molecular weight (Mn) and weight average molecular weight (Mw), derived from the chromatogram, were equal, being 2753 Da and 4291 Da, respectively, with a molecular weight polydispersity (Mw/Mn) of 1.56. The theoretical, expected molecular weight of the reaction product calculated from the monomer to initiator molar ratio was 7133 Da. The difference between the expected and experimental molecular weights suggests that part of the monomer was not converted during the applied reaction time, or that some side reactions initiated by water vapors occurred [22].

### 2.2. Particle–Polycation and Polyanion–Polycation Interactions

**Figure 5:**
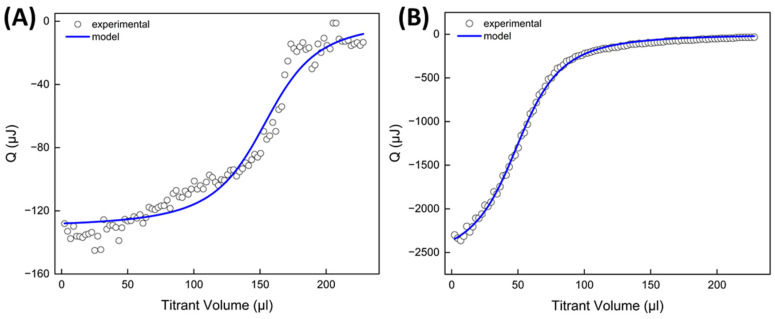
ITC titration curves reflecting interaction of polyanion HEP with polycations CHIT (**A**) and PEI (**B**) in LbL shell assembly conditions.

ITC was used to measure the heat associated with the interactions between polyelectrolytes during shell assembly, as well as the heat related to the adsorption of polycations onto LV-PCL cores. Due to the macromolecular nature of the substances investigated, an accurate determination of their molar concentrations—which is necessary for calculating thermodynamic parameters—was not feasible [23]. However, mathematical models—fitted to the experimental data—enabled determination of the binding mass ratios, in which these macromolecules interacted under shell assembly conditions (Figure 5).

**Figure 6:**
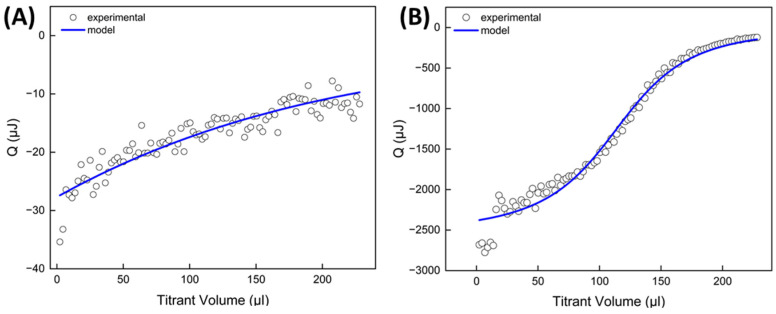
ITC titration curves reflecting interaction of LV-PVL particles with polycations CHIT (**A**) and PEI (**B**) in LbL shell assembly conditions.

For both CHIT-HEP and PEI-HEP interactions occurring in PBS at pH 4.5 and 7.4, respectively, heat release was observed during titration. The exothermic responses indicate negative enthalpy, which reflects the spontaneous ionic interactions between the amine groups of the polycations and the carboxyl and sulfonate groups of the HEP. The heat released during PEI-HEP titration was significantly higher than CHIT-HEP titration, attributed to the higher ionic charge density of PEI and its pKa value of ~7, compared to a pKa of 6.45 for CHIT [24,25]. The pH of 4.5 used during CHIT-HEP assembly favored the ionization of CHIT, which is a weak base. However, this pH is close to the 3.3 pKa of HEP’s carboxyl groups, limiting its ionization and thus potentially reducing the strength of the interaction. For the polycation, the HEP-binding mass ratio also differed between the two systems, with 0.78 for CHIT and 0.26 for PEI, likely due to the lower charge density in CHIT molecules. Additionally, the high molecular weight of CHIT may lead to a coiled conformation, limiting interactions primarily to externally charged groups, which is consistent with the greater thickness of the CHIT/HEP multilayers observed in DLS experiments [26]. Similar observations were made when LV-PCL cores were titrated with polycation solutions under shell assembly conditions (Figure 6).

The surface charge of polyester particles in aqueous suspension depends on the polymer chain’s chemical composition and terminal groups, which can be controlled during polymerization. The slight negative charge of LV-PCL particles likely arises from free hydroxyl and carboxyl groups on hydrolyzed PCL chains, as well as carbonyl oxygens in ester groups and LV moieties acting as hydrogen bond acceptors [27,28]. Consequently, the interaction between the core surface and polycationic macromolecules such as CHIT and PEI resulted in exothermic effects of a comparable magnitude to those observed with HEP. The quantity of polycation adsorbed as the initial layer per mg of core material was 0.072 mg for PEI and 0.065 mg for CHIT. This suggests a strong adsorption of a thin PEI layer, mediated by multiple interaction sites per macromolecule, and a thicker, more loosely bound layer of CHIT in a coiled conformation. These findings align with the DLS data, which showed changes in the hydrodynamic diameter during shell assembly.

### 2.3. Core-Shell Particle Characterization

**Figure 7:**
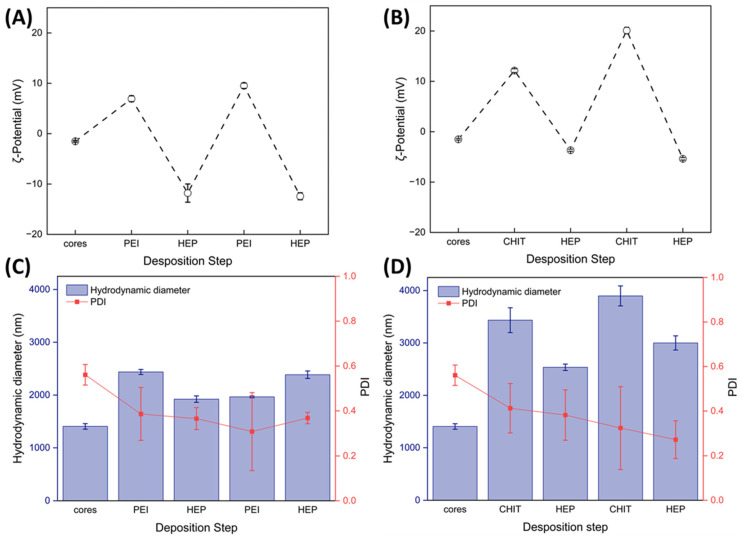
Changes in particle electrokinetic potential and hydrodynamic diameters during the assembly of (PEI/HEP)_2_ shells (**A**,**C**) and (CHIT/HEP)_2_ shells (**B**,**D**) on LV-PCL microparticles. Error bars represent standard deviations calculated from three independent measurements.

Due to the absence of a drug incorporation step during the preparation of LV-PCL particles, core fabrication via the ESE method is straightforward. Covalent drug-binding to the polymer chain in the macromolecular prodrug ensures uniform drug distribution within the polymer matrix, with drug content determined by the molecular weight of LV-PCL. The resulting microparticles served as cores for CHIT/HEP and PEI/HEP multilayer assembly. The properties of the cores and shell formation were investigated through ζ-potential and Hd measurements conducted at each step of the polyelectrolyte multilayer deposition (Figure 7).

**Figure 8:**
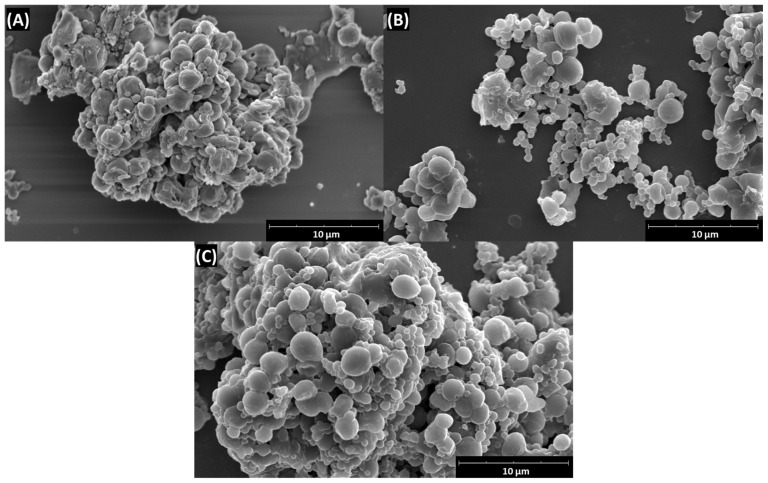
SEM micrographs of LV-PCL microparticles (**A**) and their modified variants with (CHI/HEP)_2_ shells (**B**) and (PEI/HEP)_2_ shells (**C**).

Alternating changes in ζ-potential, observed in both shell variants after the deposition of each polycation and polyanion layer, confirmed the adsorption of charged polymers on the particle surface (Figure 7A,B). The intensity of electrokinetic potential fluctuations was comparable to those observed during the polyelectrolyte multilayer deposition on PCL cores fabricated via the double emulsion solvent evaporation method [29]. The more pronounced negative ζ-potential during the PEI/HEP multilayer deposition, and the high positive values in the CHIT/HEP system, occurred primarily due to the different pHs applied during the deposition. In both coating variants, coated particles exhibited a significantly larger Hd compared to uncoated cores (*p* < 0.001, Student’s *t*-test), with an approximate shell thicknesses of 490 nm for PEI/HEP tetralayers and 797 nm for CHIT/HEP tetralayers. The greater thickness of the CHIT-based shell is likely due to its higher molecular weight of 200 kDa compared to the 25 kDa for employed PEI. Variations in Hd during both shell assembly processes, particularly with CHIT/HEP, indicate a dynamic nature of the process, involving concurrent adsorption and desorption [30]. The observed decrease in the hydrodynamic diameter PDI after the deposition of each successive layer is likely attributed to the increase in the absolute value of the ζ-potential, with a statistically significant difference observed between uncoated cores and tetralayer-coated particles (*p* < 0.01, Student’s *t*-test). The surface charge induced repulsive forces, leading to reduced aggregation and stabilization of colloidal suspension. The SEM visualization of all particle variants did not reveal substantial differences in the size of coated particles (Figure 8B,C), in comparison to cores (Figure 8A).

This is most probably due to the dehydrated state of shells in vacuum conditions, which largely differ from the native, swollen state, under the aqueous conditions in which Hds were measured [31]. Nonetheless, LV-PCL cores exhibited a more developed surface structure, with visible irregularities, in contrast to the smoother surface of CHIT/HEP- and PEI/HEP-coated particles.

### 2.4. Release Study

**Figure 9:**
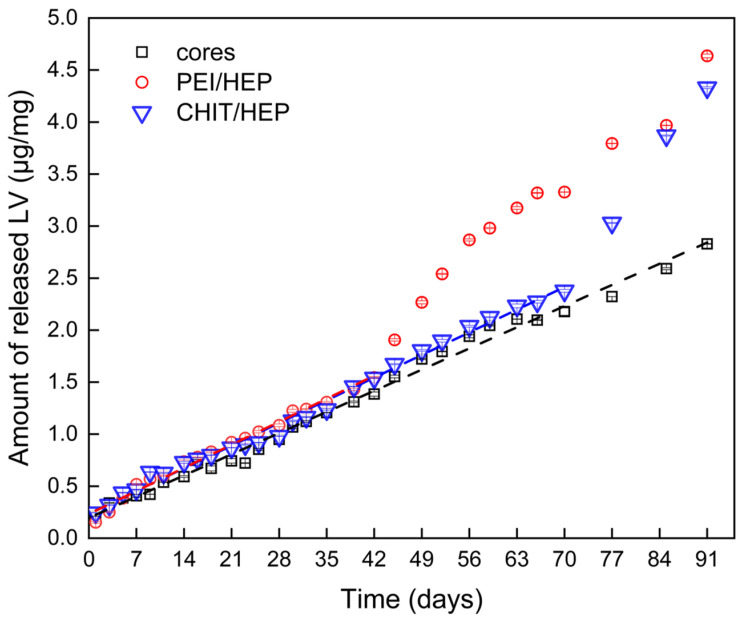
Release profiles of lamivudine (LV) from non-modified LV-PCL microparticles (black squares), (CHIT/HEP)_2_-coated LV-PCL cores (blue triangles), and (PEI/HEP)_2_-coated LV-PCL cores (red circles), along with fitted linear functions (dashed lines). The y-axis represents the mass of released LV per mg of microparticles.

Due to the high water solubility of LV, particulate systems designed for its delivery typically release the incorporated drug within hours, depending on the carrier type and formulation parameters. Most reported systems, including polyester-based nanoparticles, exhibit a well-defined burst release or bimodal release profiles [32,33,34]. In contrast, the use of an LV-PCL macromolecular prodrug resulted in a release profile that followed zero-order kinetics, as indicated by R^2^ values > 0.99 for linear functions fitted to data for 91 days in the case of uncoated cores, 71 for CHIT/HEP variant, and 42 days for the PEI/HEP variant (Figure 9).

The lack of burst release confirms that the entire incorporated dosage of LV was covalently bound to PCL chains, with no free drug present as an impurity after the synthesis process. A minor initial release of LV, ranging from 0.15 to 0.25 µg/mL, was observed on the first day of the experiment in all investigated variants, presumably due to the hydrolytic detachment of LV molecules present on the particle surface. Due to its hydrophobicity and high crystallinity, PCL is known for its slow degradation rate, with resorption of PCL-based implantable devices occurring after 2 to 3 years [35]. In the case of PCL-based particles, the higher surface area facilitates hydrolysis, accelerating the degradation rate and causing significant changes in polymer properties within weeks after exposure to physiological-like conditions [36]. Tracing the LV detachment from low molecular weight LV-PCL conjugates offers a unique method for tracking particle degradation, confirming ester bond cleavage from the first hours after particle exposure to aqueous media. For 42 days of exposition to release media, the linear release profile in all particle variants indicates a surface erosion mechanism of degradation, without signs of autocatalyzed degradation from accumulated acidic degradants within the particles. The linear stage release rate constants of 0.0311, 0.0316, and 0.0291 for CHIT/HEP, PEI/HEP, and uncoated cores, respectively, show a slightly faster release rate from coated particles. However, the differences between the fitted functions were not statistically significant, as indicated by ANOVA/Tukey tests, with the lowest *p*-value of 0.68 for the comparison between uncoated cores and particles with PEI/HEP tetralayers. This suggests that the investigated LbL shells did not act as an efficient diffusion barrier for drug release. The slight differences in release rate may be attributed to the colloid-stabilizing effect of LbL coatings, which prevented particle aggregation and, consequently, a reduction in release surface area.

For PEI/HEP-coated particles, a distinct release behavior was observed, characterized by a notable increase in the release rate after day 42 of the experiment. A similar increase in release rate and deviation from the linear release regime was observed for CHIT/HEP-coated particles after day 70 of the release experiment. The resulting bimodal release profiles are consistent with previously documented autocatalytic degradation patterns in polyester particles, where the accelerated release is correlated with a measured decrease in molecular weight [37,38]. The polyelectrolyte layers may have facilitated enhanced prodrug degradation within the particles, potentially catalyzed by the accumulation of acidic degradation products, leading to a delayed increase in the drug release rate during the later stages of the experiment. The notable differences in the time points at which changes in the release regime occurred highlight the influence of the polycation type on this phenomenon. Overall, the obtained release profiles differed significantly from those observed in systems where LV was incorporated into polyester microparticles via traditional approaches [39].

The long-term release of small LV doses from the investigated system suggests its potential application in local, prophylactic delivery of antiretroviral drugs, such as in the prevention of HIV infection [40]. Low doses of slowly released LV are sufficient to provide a local antiviral effect, which is observed at nanomolar concentrations, without the risk of systemic side effects [41]. This underscores the potential use of the described system as a component embedded in intraurethral implantable devices [42].

## 3. Materials and Methods

### 3.1. Materials

The following chemicals and materials were used in this study: ε-caprolactone (purity 97%, Sigma Aldrich, Darmstadt, Germany), lamivudine (secondary pharmaceutical standard, purity 100%, Sigma Aldrich, Darmstadt, Germany), tin 2-ethylhexanoate (purity of 92.5 to 100% Sigma Aldrich, Darmstadt, Germany), poly(vinyl alcohol) (31 kDa, degree of hydrolysis, 86.7 to 88.7%, Roth, Zielona Góra, Poland), dichloromethane (purity 98.5%, Chempur, Piekary Śląskie, Poland), tetrahydrofuran (purity 99.8%, Chempur, Piekary Śląskie, Poland), methanol (purity 99.9%, Chempur, Piekary Śląskie, Poland), chloroform (purity 99.9%, Chempur, Piekary Śląskie, Poland), ammonium acetate (purity of 97 to 100%, Chempur, Piekary Śląskie, Poland), glacial acetic acid (purity 99.5%, Chempur, Piekary Śląskie, Poland), GPC polystyrene standards (analytical standard grade, Sigma Aldrich, Darmstadt, Germany), phosphate saline buffer (0.01 M phosphate buffer, 0.0027 M potassium chloride and 0.137 M sodium chloride, pH 7.4, purity 99.9%, Sigma Aldrich, Darmstadt, Germany), chitosan (Mw~200.000, degree of deacetylation ≥90%, Pol-Aura, Olsztyn, Poland), polyethyleneimine branched (Mw~25.000, Sigma Aldrich), and heparin sodium salt (≥180 IU/mg, Pol-Aura, Olsztyn, Poland).

### 3.2. Lamivudine—Poly(ε-caprolactone) Prodrug Synthesis

LV-initiated ring-opening polymerization of ε-CL was conducted in bulk, according to a previously reported protocol. ε-CL and LV were preheated to 100 °C under a dry nitrogen atmosphere, in a round bottom flask equipped with a magnetic stirrer and reflux condenser. Subsequently, the SO serving, as the reaction catalyst, was introduced to the reaction vessel and polymerization was carried out for 5 h. The ε-CL:LV:SO reactant molar ratio was 1:0.016:0.0023. The obtained crude product was dissolved in dichloromethane, precipitated with cold methanol, dried and stored under vacuum.

### 3.3. Electrospray Time-of-Flight Mass Spectrometry (ESI-ToF MS)

The mass spectra of the synthesized macromolecular prodrug were obtained using a micrOTOF-Q mass spectrometer (Bruker Daltonics, Bremen, Germany). The sample, dissolved in chloroform, was ionized through electrospraying. Spectra were collected in positive ionization mode with the following settings: a capillary voltage of 3500 V, a nebulizer pressure of 1.5 bar, a dry gas flow rate of 8 L/min, and a dry temperature of 180 °C. The obtained data were processed using the Compass DataAnalysis 4.2 software package (Bruker, Bremen, Germany).

### 3.4. Gel Permeation Chromatography (GPC)

GPC chromatograms were obtained using a Dionex Ultimate 3000 HPLC system (Thermo Scientific, Waltham, MA, USA), equipped with a Phenogel 10^3^ Å column (Phenomenex, Torrance, CA, USA) and a refractive index detector. Chromatographic separation was performed at room temperature, with tetrahydrofuran as the mobile phase, with 1 mL/min flow. The molecular weight of the synthesized product was calculated based on a linear polystyrene standard calibration curve consisting of eleven points in the 1 to 96 kDa molecular weight range.

### 3.5. Isothermal Titration Calorimetry (ITC)

Interactions between polyelectrolytes under conditions mimicking LbL shell assembly were investigated using a Nano ITC calorimeter (TA Instruments, New Castle, DE, USA). For the polyelectrolyte interaction analysis, 0.6 mg/mL HEP solutions in PBS at pH 7.4 and 4.5 were titrated with 3.0 mg/mL PEI and 3.0 mg/mL CHIT solutions, respectively. In experiments evaluating polycation adsorption on the particle surface, 5 mg/mL particle suspension was titrated with the same solutions as in the polyelectrolyte interaction experiments. In each experiment, the analyte volume was 1.0 mL, and the titrant volume was 0.25 mL. A total of 109 injections, each 2.85 µL, were introduced at 180-s intervals. The titrations were carried out at 25 °C with a stirring rate of 300 rpm. The experimental data were fitted to a one-binding site model using the NanoAnalyze software (v. 3.1.2, TA Instruments, New Castle, DE, USA).

### 3.6. Core-Shell Particle Preparation

Particle cores were formulated using the emulsion solvent evaporation (ESE) method. An *o*/*w* emulsion, composed of a 20 mg/mL prodrug solution in dichloromethane and a 0.5% *w*/*v* aqueous polyvinyl alcohol solution, was prepared using a high-speed rotor-stator homogenizer (Ingenieurbüro CAT, Ballrechten-Dottingen, Germany), operating at 17,000 rpm for 10 min. The oil phase of the emulsion was then evaporated using a rotary evaporator at 30 °C under a 90 kPa vacuum for 25 min. The resulting solid prodrug particles were centrifuged for 10 min at 4227 rcf and washed twice with deionized water [43]. The LbL dip-coating procedure was conducted using 1.0 mg/mL solutions of CHIT, HEP, and PEI in PBS, with pH 7.4 for the PEI/HEP and pH 4.5 for the CHIT/HEP layers. For each assembly type, 10 mL of a 3 mg/mL core suspension in the appropriate PBS was prepared, and the polyelectrolyte solution was added to achieve the final polyelectrolyte concentration. Each 10 min deposition step was conducted on a rotary shaker, followed by 10 min of centrifugation at 3074 rcf and a 5 min rinsing step in PBS. Alternate deposition of polyelectrolytes continued until (PEI/HEP)_2_ and (CHIT/HEP)_2_ tetralayers were formed.

### 3.7. Particle Characterization

DLS measurements were performed on a Zetasizer Nano apparatus (Malvern, Worcestershire, UK) to confirm the deposition of each polyelectrolyte layer during shell assembly. The zeta-potential (ζ potential) and hydrodynamic diameters (Hd) were measured in PBS with a pH of 7.4 for PEI/HEP shells and PBS with a pH of 4.5 for CHIT/HEP shells. ζ-potential measurements were conducted in polycarbonate folded capillary cells with embedded gold-plated copper electrodes, while Hd measurements were performed in polystyrene cuvettes. Each sample was measured in triplicate, and the values were expressed as mean ± SD. The statistical significance of differences between the selected PDI and Hd values was evaluated using the Student’s *t*-test. Particle morphology was visualized using a scanning electron microscopy (SEM) on a Phenom Pro scanning electron microscope (Thermo Scientific, Waltham, MA, USA) at 15,000× magnification, utilizing 10 kV acceleration voltage in the secondary electron detection mode. Prior to visualization, particles were sputter-coated with a 5 nm thick gold layer.

### 3.8. Release Study and High-performance Liquid Chromatography

An LV release from three variants of the obtained particles was conducted in PBS (pH 7.4). The particles were resuspended in the release medium at a concentration of 1 mg/mL and incubated at 37 °C on an orbital shaker set to 200 rpm for 91 days. At selected time points, 0.5 mL samples of the particle suspension were centrifuged for 10 min at 3348 rcf, and 0.2 mL aliquots of the supernatants were collected and stored in a freezer until further analysis. The volume of collected samples was replenished to 0.5 mL, and the remaining particles were resuspended and transferred back to the release vessels. The concentrations of LV in the samples were measured using the pharmacopoeial high-performance liquid chromatography (HPLC) method. The analysis was performed on a Hitachi Primaide HPLC system (Hitachi HTA, Schaumburg, IL, USA) equipped with a Primaide 1410 UV detector and a Purospher STAR RP-18 column (5 µm; 250 × 4.6 mm) (Merck Millipore, Burlington, MA, USA). The mobile phase consisted of 0.025 M ammonium acetate solution, with the pH adjusted to 3.8 ± 0.2 using acetic acid and methanol in a ratio of 95:5. Chromatographic separation was carried out at 35 °C with a 1 mL/min flow rate, and detection was performed at a wavelength of 277 nm. LV concentrations were calculated based on a linear calibration curve constructed from 7 concentrations, ranging from 0.775 to 49.6 µg/mL, with an R^2^ > 0.999. Zero-order kinetic models were fitted to the experimental data and statistically compared using the least squares method, ANOVA, and Tukey tests in Statistica software (TIBCO Software Inc., Palo Alto, CA, USA).

## 4. Conclusions

The results of this study demonstrate that the design of macromolecular lamivudine prodrug microparticles, combined with LbL polyelectrolyte coatings, offers a promising strategy for achieving prolonged and controlled drug release. The covalent attachment of lamivudine to PCL chains (4291 Da) ensured a stable and predictable release profile that closely followed zero-order kinetics for 42, 70, and 91 days for PEI/HEP, CHIT/HEP, and uncoated particles, respectively. This confirms that the entire drug load was covalently integrated into the polymer matrix, effectively avoiding the significant burst release typically observed with free drug-loaded particulate systems. Although the role of LbL coatings as diffusion barriers was not conclusively established, the results suggest that these coatings contributed to the colloidal stability of the particles, as evidenced by the high ζ-potential values. Additionally, the bimodal release observed in coated particles suggests that the presence of LbL shells may promote autocatalytic degradation of the prodrug within the particles. The steady release of 0.03 µg of lamivudine per milligram of uncoated cores per day can be modified through the incorporation of optimized LbL coatings. This study also highlights the utility of ITC in investigating the thermodynamics of polyelectrolyte assembly, revealing the electrostatic nature of interactions characterized by negative enthalpy and the mass ratios of polyelectrolytes involved in LbL shell formation. The prolonged, steady release of lamivudine underscores the potential of these systems for localized drug delivery, particularly in applications requiring the extended release of controlled drug concentrations, such as the prophylactic treatment of chronic viral infections.

## Data Availability

Dataset available on request from the authors.
